# Measuring change in health status of older adults at the population level: The transition probability model

**DOI:** 10.1186/1472-6963-10-306

**Published:** 2010-11-09

**Authors:** Rahim Moineddin, Jason X Nie, Li Wang, C Shawn Tracy, Ross EG Upshur

**Affiliations:** 1 Institute for Clinical Evaluative Sciences, Toronto, Canada; 2 Department of Family and Community Medicine, Faculty of Medicine, University of Toronto, Toronto, Canada; 3 Primary Care Research Unit, Sunnybrook Health Sciences Centre, Toronto, Canada; 4 University of Toronto Joint Centre for Bioethics, Toronto, Canada; 5 York University, School of Kinesiology and Health Science, Toronto Canada; 6 Dalla Lana School of Public Health, University of Toronto, Toronto, Canada

## Abstract

**Background:**

The current demographic transition will lead to increasing demands on health services. However, debate exists as to the role age plays relative to co-morbidity in terms of health services utilization. While age has been identified as a critical factor in health services utilization, health services utilization is not simply an outcome of ill health, nor is it an inevitable outcome of aging. Most data on health service utilization studies assess utilization at one point in time, and does not examine transitions in health service utilization. We sought to measure health services utilization and to investigate patterns in the transition of levels of utilization and outcomes associated with different levels of utilization.

**Methods:**

We conducted a population-based retrospective cohort study of all Ontario residents aged 65+ eligible for public healthcare coverage from January 1998-December 2006. The main outcome measure was total number of utilization events. The total is computed by summing, on a per annum basis, the number of family physician visits, specialist visits, Emergency Department visits, drug claims, lab claims, X-rays, CT scans, MRI scans, and inpatient admissions. Three categories of utilization were created: low, moderate, and high.

**Results:**

There is heterogeneity in health services utilization across the late lifespan. Utilization increased consistently in the 9-year study period. The probability of remaining at the high utilization category when the person was in the high category the previous year was more than 0.70 for both males and females and for all age groups. Overall healthcare utilization increases more rapidly among the high users compared to the low users. There was negligible probability for moving from high to low utilization category. Probability of death increased exponentially as age increased. Older adults in the low utilization category had the lowest probability of death. The number of male nonagenarians increased more rapidly than female nonagenarians.

**Conclusion:**

There are measurable and identifiable differences in the patterns of health services utilization among older adults. This data will permit clinicians and policy makers to tailor interventions appropriate to the risk class of patients.

## Background

There is a demographic transition occurring in the developed world. An aging population creates new demands on health services [1]. Health service utilization increases, on average, across the life course, particularly in the late life course years [2,3]. It is expected that the baby boom generation will result in a large cohort of aging individuals with different health services utilization patterns than previous generations.

Debate exists in the health services literature concerning the role age plays relative to co-morbid illness in terms of health services utilization. Is the primary explanation for increases of utilization seen in the late life course related to age or the worsening of health status and the accumulation of chronic diseases? Many studies have identified age as a critical factor in health services utilization, These studies have failed to emphasize or acknowledge that i) health services utilization is not simply an outcome of ill health and ii) health services utilization is not an inevitable outcome of aging [4]. Factors such as availability of services, as well as social and other contextual aspects may have direct and indirect influences on the use of health services [5]. For example, it has been shown that improved nutrition and exercise have the potential to improve health and to reduce health services utilization by older adults [4].

It is critical to understand, at both the clinical and population level the determinants of the transition in health service utilization in the late life course. Transitions, in this study, refer to changes in the pattern of health service utilization over time, particularly in relation to the number of health service utilization events an individual experiences over a set period of time. It is likely that this is a dynamic process influenced by a large number of factors including changes in health status. Transitions in health services utilization can be understood as events in a dynamic system occurring with measurable probability. These probabilities may vary by the age and gender of the individual.

Most studies on health service utilization in the late life course assess utilization at one point in time and hence do not capture the dynamics of transitions in utilization of health services. The existence of large administrative databases in the province of Ontario, including the healthcare records of more than 6 million residents age 65 and older, over a period of many years, provided the opportunity to conduct linked population-based studies by age and sex to study in detail healthcare utilization at the population level over time. This unique opportunity allows us to understand better the transitions in health service utilization while testing epidemiological methods that may result in clinically relevant tools.

To the best of our knowledge, no previous study has measured the transition between levels of health service utilization among the population 65 years of age and older using population-based data. The objectives of this study are:

1.) To describe trends in health service utilization by age and gender in the population of Ontario over the age of 65.

2.) To measure the transition probability in the level of health service utilization for people 65 years and older for a nine year period, as well as the relationship of the transition probability to age and gender.

3.) To measure trends over time for the transition probabilities for different age groups and genders.

4.) To measure the association between the level of health service utilization and mortality.

## Methods

### Study cohort

We conducted a population-based longitudinal cohort study to examine the health service utilization events over a 9-year period (January 1, 1998 to December 31, 2006). All Ontario residents 65 years of age or older who were eligible for the Ontario Health Insurance Plan (OHIP) and who had at least one contact with healthcare system were included in the study. OHIP includes all fee-for-service physician claims. Doctors and patients enrolled in alternative payment plans, who are still in the minority, were not captured, nor were utilization events within other sectors of the Ontario health care system not covered by OHIP. (e.g., home care, physiotherapy, etc.). Services provided to patients without valid OHIP numbers and/or out-of-province patients were excluded from the analysis. If a person did not have any contact with the healthcare system for two consecutive years or if they were dead, he/she was excluded from the follow up after his/her last date of contact. Patient health card numbers were used to identify age and sex from the Ontario Registered Persons Database. Subjects were grouped by sex and 5-year age intervals.

We quantified healthcare utilizations by adding the number of family physician (FP) or general practitioner (GP) visits, number of specialist (SP) visits, number of inpatient admissions, number of X-Ray, CT, and MRI tests ordered, number of emergency department (ED) visits, and number of drug claims over period of one year. This index number will be referred to as the number of events. This index measures the level of individual health care utilization.

### Administrative data sources

Four data sources were used to conduct the analysis: 1) Ontario Health Insurance Plan (OHIP) database, 2) Ontario Drug Benefit (ODB) prescription claims database, 3) Canadian Institute for Health Information (CIHI) database, and 4) Ontario Registered Persons Database (RPDB).

The OHIP database covers all claims made by fee-for-service physicians, community-based laboratories, and radiological facilities paid by the Ontario Ministry of Health.

The ODB prescription claims database contains information on outpatient prescription drug use and costs for all residents over 65 years of age in Ontario. Ontario residents may fill prescriptions at any pharmacy within the province. The CIHI discharge abstract database contains clinical administrative data relating to the health care services provided to patients by all hospital facilities in Ontario. The RPDB contains data on mortality.

All the databases allow the linkage of individuals across each database through the use of a confidential, scrambled health card number. No personal identifiers are available with these administrative data.

### Variables

All OHIP fee codes were reviewed to identify consultations, examinations, and procedures. Office visits to FP/GP or specialists were based on the physician's specialty and visits located in the physician's office. For all physician types, multiple fee codes billed by the same physician on the same day were counted as one visit. Emergency room visits were extracted for the entire study population using an algorithm developed at the Institute for Clinical Evaluative Sciences (ICES). Fee codes were also used to identify X-rays and MRI and CT scans, limited to one scan per patient per day. All laboratory procedures or tests performed during our study period were also included in the analysis.

The ODB prescription claims database was used to determine number of drug claims. Data from the CIHI were used to determine the number of inpatient admissions. Our measure of total utilization events was computed by summing all utilization events occurring during the study period (i.e., number of FP/GP visits, SP visits, ER visits, inpatient admissions, drug claims, lab claims, X-rays, CT scans, and MRI scans).

### Statistical analysis

We used Cronbach's Alpha to examine the internal consistency within our measures. Generally, Cronbach's Alpha of size 0.7 and above is acceptable. Each person was followed for up to nine years and was classified in each year of follow up as a low user if the total number of events was lower than the first quartile (Q1), a moderate user if total number of events was less than the third quartile (Q3), and a high user otherwise. We did not change these groupings for different genders and age groups in order to keep the measures simple and consistent.

Transition probabilities are the probability of moving from one utilization group to another within one year. Mortality probabilities are the probability of death within one year. We estimated the transition probabilities for each gender by age group by calculating the ratio of the number of transitions between utilization groups.

The occurrence of events over time in medical studies can be described as a finite state stochastic process with states representing health status or level of health service utilization [6]. Previous studies [7] have used transition probabilities to dependency, institutionalization, and death among older persons over a decade, as well as to monitor the changing trends in health status of an older person [8].

All analyses were done using SAS software version 9.1 (SAS Institute, Inc., Cary, NC). This study was approved by the Research Ethics Board of Sunnybrook Health Sciences Centre.

## Results

### Trends in health services utilization

The yearly (1998 to 2006) estimated Cronbach Alpha for number of FP/GP visits, SP visits, ER visits, inpatient admissions, drug claims, lab claims, X-rays, CT scans, and MRI scans ranged from 0.74 to 0.76 which confirms the internal consistency for the measure of total utilization events as an appropriate index.

Table 1 shows the number of people aged 65 and over who were eligible for universal health insurance and had at least one contact with healthcare system, by sex and 5-year age intervals. Over the 9-year period, the number of people within the same age group increased with each successive year. In each year, there were fewer numbers of people at the oldest age group in comparison with younger age groups. There were more females than males in all age groups. The number of male nonagenarians who had contact with the health care system increased more rapidly than female nonagenarians. Overall, males aged 90 and over who used the health care system increased from 7,599 to 28,560 (a 3.7 fold increase) while females age 90 and over increased from 24,747 to 69,431 (a 2.8 fold increase).

**Table 1:** Number of elderly patients by age and sex, Ontario, Canada, 1998-2006

AGE GRP	1998		1999		2000		2001		2002		2003		2004		2005		2006	
	F	M	F	M	F	M	F	M	F	M	F	M	F	M	F	M	F	M
**65-69**	229,062	206,786	231,920	211,646	232,858	213,856	235,033	217,481	234,634	217,328	237,563	219,596	239,681	221,301	242,621	223,518	244,229	223,933
**70-74**	211,941	167,776	214,869	176,672	219,825	186,909	223,188	194,621	223,639	196,335	222,453	197,206	222,451	197,772	221,120	196,551	221,178	197,249
**75-79**	168,333	113,291	183,171	126,246	192,445	136,833	200,355	147,382	201,284	151,545	202,070	155,249	200,936	158,288	202,091	162,887	201,931	165,699
**80-84**	99,891	55,869	108,708	63,369	122,796	74,671	137,464	86,770	146,854	93,298	155,355	99,928	163,433	105,440	166,760	109,280	169,061	113,130
**85-89**	53,950	23,700	63,922	29,762	73,377	35,940	83,080	42,802	84,351	43,978	86,157	45,353	88,251	47,062	95,319	51,831	102,342	56,368
**90-94**	19,582	6,401	24,667	8,557	30,917	11,456	37,416	14,597	39,637	15,829	41,863	17,127	44,917	18,724	47,039	20,089	48,881	21,576
**95-99**	4,477	1,060	6,246	1,551	8,494	2,276	11,473	3,300	12,220	3,644	13,098	4,075	13,797	4,472	15,053	5,071	15,962	5,523
**100+**	688	138	1,032	224	1,582	345	2,398	525	2,674	603	3,085	760	3,498	915	4,006	1,164	4,588	1,461

The classification cut points for the transition probability analysis (Q1: first quartile, and Q3: third quartile) are given in Table 2. Under the null hypothesis that age, sex, and time have no impact on the total number of events, we expect to observe 25% of the people being in the low users group, 50% in the moderate and 25% in the high users group. Any departure from the thresholds of 25% and 50% indicates the impact of age while year and sex are fixed, sex while year and age are fixed, or time while sex and age are fixed. We can similarly assess the interaction among these factors. The first quartile increased from 19 to 26, an increase of a half-event in each year, and the third quartile increased from 61 to 81, two-event increase per year, over the period of 9 years.

**Table 2:** The first and third quartile for total events per person by year used for the classification of cut points for low-, moderate- and high- users among patients aged 65+ in Ontario, Canada, 1998-2006

Year	First quartile	Third quartile
1998	20	63
1999	20	63
2000	21	68
2001	22	70
2002	24	73
2003	24	76
2004	25	78
2005	26	80
2006	26	81

### Low/moderate/high users of health services

The trends over time for the percent of female and male patients among different utilization categories for all age groups are given in Table 3. There is a decline in the percentage of males and females in the low and moderate categories as age increases, while there is an increase in the percentage of males and females in the high category. Over the 9-year period, there is an increase in the percentage of males and females in the low category for people age 65 to 74, while there is a decline over time for people 80 and over in the low category. There is a clear decline in the percentage of male and female high users over time for people 65 to 79, and an increase over time for people 80 years and over.

**Table 3:** Proportion of low-, moderate-, and high-users among patients aged 65+ in Ontario, Canada, 1998-2006

AGE GROUP	USER GROUP	1998		1999		2000		2001		2002		2003		2004		2005		2006	
		F	M	F	M	F	M	F	M	F	M	F	M	F	M	F	M	F	M
**65-69**	**Low**	32.1	37.8	32.7	38.9	33.6	40.0	33.5	39.6	33.9	39.8	35.8	41.4	35.0	40.2	35.6	40.5	36.6	41.1
	**Moderate**	49.4	46.7	49.2	45.9	49.4	45.6	50.8	46.5	50.5	46.4	49.1	45.2	50.5	46.8	50.1	46.5	49.5	46.2
	**High**	18.5	15.6	18.1	15.3	17.0	14.4	15.7	13.8	15.7	13.8	15.1	13.4	14.5	13.1	14.3	13.1	13.9	12.7
																			
**70-74**	**Low**	23.6	27.8	24.4	28.7	24.6	29.3	24.3	28.6	24.1	28.7	25.7	29.8	24.6	28.6	24.8	28.5	25.1	28.8
	**Moderate**	50.9	50.0	50.9	49.4	51.9	49.5	53.4	50.9	53.5	50.9	52.5	50.0	54.4	51.7	54.2	51.9	54.2	51.8
	**High**	25.5	22.2	24.7	21.9	23.5	21.2	22.3	20.5	22.4	20.3	21.8	20.2	21.1	19.7	21.0	19.6	20.7	19.4
																			
**75-79**	**Low**	20.3	23.4	20.7	24.1	20.6	23.9	20.1	23.3	19.9	23.1	21.0	24.1	19.9	22.6	20.0	22.4	20.1	22.4
	**Moderate**	50.2	50.0	50.1	49.1	50.8	49.8	52.4	50.8	52.3	50.8	51.6	50.1	53.5	52.0	53.4	52.1	53.4	52.3
	**High**	29.4	26.5	29.3	26.8	28.6	26.3	27.5	25.9	27.8	26.1	27.3	25.8	26.6	25.4	26.6	25.5	26.5	25.2
																			
**80-84**	**Low**	17.9	21.6	18.5	21.4	17.9	20.8	17.4	19.7	17.4	19.8	18.1	20.5	16.8	18.9	16.9	18.7	16.8	18.7
	**Moderate**	49.4	49.3	48.9	48.6	49.4	48.8	50.5	49.9	50.2	49.4	49.4	48.8	50.5	50.1	50.2	50.0	50.2	50.0
	**High**	32.7	29.1	32.7	30.0	32.7	30.4	32.1	30.4	32.5	30.8	32.5	30.8	32.6	30.9	33.0	31.2	33.0	31.2
																			
**85-89**	**Low**	16.7	21.1	17.0	20.6	16.4	19.8	15.8	18.2	15.3	17.6	16.1	18.3	14.8	17.2	14.7	16.7	14.9	16.4
	**Moderate**	47.8	48.1	47.3	47.6	47.0	47.5	47.1	47.8	45.9	47.3	44.4	46.1	45.7	46.9	44.5	46.6	44.3	46.5
	**High**	35.4	30.9	35.7	31.8	36.6	32.7	37.1	34.0	38.8	35.1	39.6	35.6	39.5	35.9	40.8	36.7	40.8	37.1
																			
**90-94**	**Low**	17.4	22.5	16.9	22.0	16.1	20.4	15.2	18.7	14.7	18.2	15.2	18.4	14.1	16.5	13.6	16.8	13.6	16.1
	**Moderate**	45.6	47.0	44.9	45.4	44.3	45.5	44.1	45.6	42.1	42.9	39.8	42.2	39.0	43.4	37.7	41.6	36.8	41.6
	**High**	37.0	30.4	38.2	32.5	39.6	34.1	40.7	35.7	43.2	39.0	45.1	39.4	46.9	40.1	48.7	41.6	49.6	42.3
																			
**95-99**	**Low**	16.4	23.3	17.6	23.4	16.2	19.2	15.7	18.3	14.7	17.3	15.1	18.7	14.0	17.8	13.7	17.0	13.5	17.8
	**Moderate**	44.8	43.2	42.3	44.5	41.7	44.5	41.4	42.4	37.9	41.0	33.5	38.7	33.3	38.2	31.2	35.3	30.3	34.5
	**High**	38.7	33.5	40.1	32.0	42.2	36.4	43.0	39.4	47.4	41.7	51.4	42.6	52.7	44.0	55.1	47.7	56.2	47.7
																			
**100+**	**Low**	19.9	45.5	19.9	32.4	17.5	27.1	15.5	20.0	16.0	24.2	16.7	20.3	16.1	20.9	16.0	22.1	15.8	19.3
	**Moderate**	43.1	30.9	40.8	36.3	43.5	40.0	43.7	45.7	38.7	36.8	33.4	34.8	32.7	31.6	29.7	28.4	26.3	26.7
	**High**	37.0	23.6	39.2	31.4	39.0	32.9	40.8	34.3	45.4	39.0	49.9	44.9	51.2	47.5	54.3	49.6	57.9	54.0

### Transition probabilities in level of utilization

Transition probabilities for males and females to move from one utilization category to another in the next year are presented in Table 4. Overall, males are more likely than females to stay in the low category in the next year when their present utilization category is low. Females are more likely than men to move to the moderate utilization category when their present utilization category is low. The probability of moving from the low to moderate category decreases as age increases. Both genders have the same probability of moving to the high utilization category in the next year when their present utilization category is low. The probability of moving from the low to high category increases as age increases. The probability of moving from the moderate to high category increases as age increases, and the probability of staying in the moderate utilization group decreases as age increases.

**Table 4:** Transition probabilities by sex and age group among patients aged 65+ in Ontario, Canada, 1998-2006

Time Transition		65-69		70-74		75-79		80-84		85-89		90-94		95-99		100+	
Index Year	Next Year	F	M	F	M	F	M	F	M	F	M	F	M	F	M	F	M
**Low Users**	*Low*	0.57	0.62	0.62	0.65	0.60	0.62	0.59	0.61	0.59	0.60	0.60	0.61	0.62	0.60	0.66	0.70
	*Moderate*	0.40	0.35	0.36	0.33	0.37	0.35	0.37	0.36	0.36	0.36	0.34	0.34	0.32	0.33	0.29	0.24
	*High*	0.03	0.03	0.02	0.02	0.02	0.03	0.03	0.03	0.05	0.04	0.06	0.06	0.07	0.07	0.06	0.06
																	
**Moderate Users**	*Low*	0.16	0.17	0.15	0.16	0.13	0.14	0.12	0.13	0.11	0.12	0.12	0.13	0.12	0.14	0.13	0.18
	*Moderate*	0.71	0.69	0.73	0.71	0.72	0.71	0.71	0.70	0.69	0.68	0.66	0.66	0.63	0.63	0.64	0.56
	*High*	0.13	0.13	0.13	0.13	0.15	0.15	0.17	0.18	0.20	0.20	0.22	0.22	0.25	0.23	0.23	0.26
																	
**High Users**	*Low*	0.01	0.01	0.01	0.01	0.01	0.01	0.01	0.01	0.01	0.01	0.01	0.01	0.01	0.01	0.01	0.02
	*Moderate*	0.28	0.29	0.27	0.28	0.25	0.25	0.22	0.23	0.18	0.20	0.14	0.18	0.13	0.15	0.14	0.15
	*High*	0.71	0.70	0.72	0.71	0.75	0.74	0.78	0.76	0.82	0.79	0.85	0.81	0.87	0.84	0.85	0.86

Males and females have approximately the same probability of staying in the moderate utilization category or moving to either the low or high utilization category when their present utilization category is moderate. Both males and females, regardless of their age, have a very low probability of moving to the low utilization category in the next year when their present utilization category is high. Males are more likely to move from the high to the moderate utilization category in one year than are females. Table 4 shows that as age increases, the chance of moving from the high to moderate category decreases while the probability of staying in the high utilization category increases.

The probability of remaining at the high category when the person is in the high category the previous year is more than 0.70 for both males and females, and for all age groups. However, the transition probabilities from the high category to the low or moderate category the next year are less than 0.02 and 0.29 respectively. The transition probabilities of moving from the moderate to the high category range from 0.13 to 0.26.

### Trends over time in transition probabilities

We examined the trends of transition probabilities from the low user category to moderate and high users, from moderate to low and high users, and from high users to moderate and low users over a period of nine years (data not shown). The probability of moving from the low to the high category for both males and females increased over time for people age 79 and over, whilst the probability of moving from the low to the high category did not change over time for both genders age 65 to 79 years. The probability of moving from the low to the moderate utilization category was fairly flat over time for both genders. The probability of moving from the moderate to the high category for both genders age 65-85 was flat over time, while it increased over time for both genders age 85 and over. The probability of moving from the moderate to the low utilization category was flat over time for females, while it decreased for males. The probability of moving from the high to the low utilization category slightly decreased over time for both genders.

### Mortality probability

**Figure 1:**
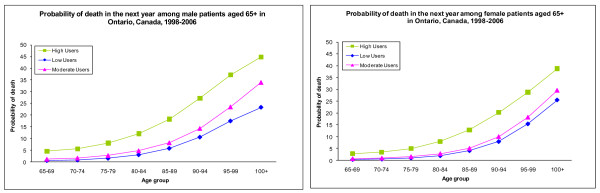
**Probability of death in the next year by sex and age group among patients aged 65+ in Ontario, Canada, 1998-2006**.

Males who were in the high category had a higher probability of death compared to females who were in the high category for all age groups (Figure 1). This difference increased from 2% to 6% as age increased. Both males and females who were in the high category were more likely to die in the next year compared to those who were in the low or moderate categories. Consistently, males were more likely to die in the next year compared to females for all age groups except those 95 and over. The mortality probability gap between males and females widened after age 70 and then narrowed after age 95. The probability of death in the next year was between 23-45% for people who managed to survive to age 100.

## Discussion

Our study, using population-level data, indicates significant heterogeneity in the level of health services utilization among older adults. Several important trends emerge over the 9 year period. The population of men aged 90 and over who had contacts with healthcare system grew faster than the population of women in the same age group. The population in the high utilization category increased more rapidly than those in the low utilization category. Our estimated transition probability models demonstrate that when a person moves to the high utilization category, it is almost impossible to transition back to the low utilization category. High users are more likely to stay in the high category than move to the moderate category. It is not clear what is driving this constant increase over time. Finally, those in the high utilization category have a higher probability of death the next year than moderate or low users.

The magnitudes of the transition probabilities from the low and moderate utilization categories to the high category are substantial and indicate high demands on patients and their families, on professional health care providers, and on the health care system itself. The increase in health service utilization in older adults has been shown to be largely associated with poorer health outcomes [9].

A large amount of utilization is associated with medications [10]. As Nie et al. show, the number of prescriptions increases substantially for people over the age of 65 in the province of Ontario [2]. Most medications are prescribed to seniors by family physicians, and the vast majority are prescribed for the management of the common chronic diseases of aging such as osteoporosis, hypertension, osteoarthritis, and cardiovascular disease [11]. A population-based study examining the level of total outpatient pharmaceutical expenditures in the province of British Columbia showed that high-cost users of prescription drugs were older, more likely to be female, had lower incomes, and had more diagnosed co-morbidities. With regard to the transition probabilities in pharmaceutical spending level, seniors in the high-cost category in 2001 had more than a 64% chance of either continuing to be in the highest decile of drug spending in 2004 or dying within the 3-year period, and were also highly unlikely to transition to spending deciles below the median in 2004 [12].

Our results show that women have higher rates of health services utilization than men in all years and in all age groups. This is consistent with a previous study that concluded gender is an important predictor of medical care use before and after removing sex-specific utilization [13].

Our data show that mortality is consistently higher in the high utilization groups of both genders, though with dramatic differences. Males in the moderate and low categories of health service utilization had a higher probability of death the next year than females in the moderate and low utilization category. However, males in the low utilization category surviving past 95 years of age demonstrated a significant drop in mortality probability. In a previous study looking at the risk of mortality among older adults over an 8-year period, the six most salient predictors of mortality were identified as the mean annual number of hospital episodes after baseline, age, female gender, non-kin social supports, body mass, and having a history of diabetes [14]. In another study looking at older women, mortality was predicted by heart disease, stroke, low iron, diabetes, cancer (non-skin), bronchitis/emphysema, and Alzheimer's disease [15]. Other factors, such as repetitive falling, have been shown to be related to an increased likelihood of hospitalization and death [16].

Our results also depict a disconcerting trend in the increase of utilization on an annual basis. The first and third quartiles showed 30% and 28% increases over the study period, indicating a progressive escalation of baseline utilization rates in these categories. This means that, on average in each year, the cut point for the low, moderate and high use categories crept upwards. However, it is unclear what benefit is conferred by steadily increased utilization. While the data do not permit commentary on the appropriateness of the increasing levels of utilization over time, they certainly point to scenarios of problematic sustainability, particularly in an era of cost containment and fiscal restraint. It is imperative that research into determining the appropriate level of utilization be conducted as well as more precise delineation of outcomes of care in advanced age.

The reason for the increases in health service utilization over time is unclear. It is not likely a function of increased physicians supply, as Ontario has a chronic shortage of family physicians. The supply of family physicians has not kept pace with population increases. The most significant increases in utilization are associated with medication claims. Thus, it seems reasonable to hypothesize that changes in clinical practice guidelines may be implicated in the increase in utilization, as many medications require ongoing laboratory monitoring and follow up. This will be a topic for future research.

While our data shows that health service utilization increases with age, there is also evidence of resiliency in the older population, in that it identifies a growing cohort surviving into the oldest age group who were more likely to have been low users of health care services initially. On the other hand, the data suggest significant challenges in sustainability. The combination of the increasing number of people age 65 and over, with increasing probabilities of moving into the high utilization category with age, and the unlikely chance of reducing healthcare services utilization indicates that healthcare utilizations may increase exponentially with attendant cost escalation. This emphasizes the overarching importance of prevention and health promotion in the years preceding age 65 and beyond. The healthier one is entering the later years, the less likely one will require health services.

There are very few published peer-reviewed studies examining the association between age and healthcare service utilization using the transition probability matrix at a population level. The present study uses a very large sample with reliable administrative database linkages for a comprehensive array of indicators of health service utilization over a long duration. The use of administrative data provides significantly better predictions of death than variables obtained from interview data [17].

There are several potential limitations with our study. The OHIP database includes only fee-for-service claims, therefore, doctors and patients enrolled in alternative payment plans, who are still in the minority, were not captured, nor were utilization events within other sectors of the Ontario health care system (e.g., home care, physiotherapy, etc.). Exclusion of these services leads to an underestimation of overall utilization of services. The Ontario Drug Benefit database includes only the prescriptions dispensed for residents of Ontario age 65 and over and thus will not capture over the counter medication usage. Thus the models no doubt underestimate total health service utilization. However, the model captures the majority of services provided under the universal health care insurance program in Ontario. As well, the models may underestimate the numbers of people in the higher utilization categories in the later years of the study. Rather than employing fixed cut points, we left the categorization of utilization levels vary by year. Had we used an absolute cut point, we would likely have had greater numbers in the higher utilization categories.

Utilization variables which were used for categorizing the residents of Ontario into one of the low, moderate, or high utilization categories were not weighted to account for the fact that a visit to the emergency department or hospital admission may reflect a more serious illness than a visit to a family physician. Also, although this study assumes independence of date, patients often schedule multiple appointments on the same day so as to minimize the total number of trips. The number of utilizations for those residents who left the province for a period of less than two years is under-counted, which could inflate the probability of moving from the high utilization category to the moderate or low category and deflate the probability of moving from the low or moderate category to the high utilization category.

Finally, the present study did not look at health service utilization of younger persons and that they too can be high users of health services.

## Conclusions

This pattern of findings highlights the need for a long-term strategy in aging with a focus on chronic disease management[18] This is especially pertinent as we have demonstrated that people entering the age of 65 as high users of health services are very likely to remain high users. The increasing number of nonagenarians indicates a pressing need to understand the health needs and preferences of individuals well past their actuarial life expectancy. We believe this study will be informative to clinicians as it will enable them to communicate to patients and caregivers about the ongoing need to plan for high rates of health services utilization. We believe this study will be of value to policy makers and health planners as it will assist in modeling need for service delivery. Future studies should focus on developing clinically relevant risk scores to aid clinicians in estimating risk of transitioning to higher levels of utilization. Analysis of mortality and its association with number of medications used would further contribute to our understanding of life course health service utilization patterns and mortality.

## Competing interests

The authors declare that they have no competing interests.

## Authors' contributions

RU and RM conceived the study. RM and LW conducted the data analysis. All authors contributed to the interpretation of the data and all authors were involved in the preparation of the manuscript. All authors have read and approved the final manuscript version. RU is the principal investigator of the study and will serve as guarantor.

## Pre-publication history

The pre-publication history for this paper can be accessed here:

http://www.biomedcentral.com/1472-6963/10/306/prepub
